# A New Tool to Assess the Economic Impact of Q Fever on Dairy Cattle Farms

**DOI:** 10.3390/ani14081166

**Published:** 2024-04-12

**Authors:** Didier Raboisson, Guillaume Lhermie, Raphael Guatteo

**Affiliations:** 1 CIRAD, UMR ASTRE, 34398 Montpellier, France; 2 ASTRE, CIRAD, INRAE, Univ Montpellier, 34090 Montpellier, France; 3 ENVT, Université de Toulouse, 31400 Toulouse, France; 4 Department of Production Animal Health, University of Calgary, Calgary, AB T2N 4Z6, Canada; 5 Oniris, INRAE, BIOEPAR, 44300 Nantes, France; raphael.guatteo@oniris-nantes.fr

**Keywords:** economics, cost, vaccine, benefits

## Abstract

**Simple Summary:**

To support decision-making in the field, a tool dedicated to Q fever for farmers and farm advisers was developed. The proposed, modified partial budgeting approach integrates a simplified yearly compartmental model and the main interactions between disorders linked to Q fever. The model concomitantly estimates the yearly burden of Q fever in herd prevaccination as well as the 3-year vaccination benefit. For herds with a moderate or high prevalence of Q fever prevaccination (>30%), a vaccination benefit was observed. The vaccine should then be seen as insurance in herds with low prevalence rates of Q fever prevaccination (≤20%).

**Abstract:**

To support farmers in their decisions related to Q fever, a dedicated economic assessment tool is developed. The present work describes the calculator, its economic rationale, and the supporting assumptions. The calculator integrates a yearly compartmental model to represent population dynamism and the main interactions between disorders linked to Q fever, especially reproductive disorders (abortion, retained foetal membranes, purulent vaginal discharge and endometritis, extra services, and calving–conception delays). The effects of the nontangible cost of the disease on human health, the welfare of the animals, and the workload of farmers were not integrated into the model. The model shows high-level sensitivity to the prevalence of Q fever in the herd prevaccination and to the costs of abortion and extra days of calving–conception intervals. Breakeven points, i.e., cost values that allow us to achieve positive vaccination benefits, are also reported. For herds with moderate or high prevalence rates of Q fever prevaccination (>30%), a vaccination benefit is observed. The vaccine should be considered a type of insurance in herds with low prevalence rates of Q fever prevaccination (≤20%). The calculator was developed to aid decision-making at the farm level, and no conclusion can be extrapolated as a generic trend based on the present work.

## 1. Introduction

*Coxiella burnetii* is a zoonotic bacterium with a worldwide distribution that is responsible for Q fever. It can be asymptomatic in humans, or it can present with flu-like symptoms [1]; additionally, this bacterium can affect other species [1,2,3]. The average overall animal prevalence, interherd prevalence, and intraherd prevalence of Q fever in cattle are reported to be 20%, 40%, and 20%, respectively [4]. In France, a 2017 seroprevalence survey [5] reported a herd-level prevalence of 36% for cattle.

The clinical impact of Q fever differs among ruminant species; for instance, abortion related to Q fever is epidemic in goats but more endemic in cattle. In cattle, *Coxiella burnetii* infection has been found to be responsible for other reproductive disorders, such as endometritis [3,6,7,8], retained fetal membranes (RFMs) [9,10,11] and subfertility [12,13,14]. An inactivated *Coxiella burnetii* phase-I vaccine (Coxevac^®^, Ceva Santé Animale, Libourne, France) has been authorized for cattle, goats, and sheep and is commercially available in many countries. This vaccine contributes to mitigating the zoonotic risk of Q fever through a reduction in shedding via milk, faeces, and vaginal discharge, especially around parturition, as observed by Schulze [15].

No literature is available on the economics of Q fever in cattle and the potential economic benefits for the farmer of vaccination at the herd level. Previous studies have focused on the zoonotic impact of Q fever globally [16,17,18], for instance, by assessing the cost of intervention in the animal sector to prevent human infection [19], the cost-effectiveness of chronic Q fever screening in humans [20], and the economics of Q fever vaccination for agricultural industry workers [21]. Farmers face many challenges on their farms and have to make decisions on Q fever management based on either sanitary (animal or public health issues) or economic concerns. To support farmers and their veterinarians in this decision, a Q fever economic assessment calculator is created. Performing the abovementioned studies is challenging since the association between Q fever and health or production disorders is imprecisely quantified, and reproduction is the result of multiple factors, making it difficult to estimate the fraction of reproduction attributable to Q fever. The economic assessment of the total impact of a disease or of the potential benefits of mitigation measures such as vaccination requires precise knowledge of the epidemiology of the disease [22]. The lack of precise data on the epidemiologic impact of Q fever prevents any accurate economic assessment and may explain the lack of publications on the economics of Q fever despite the potentially high impact of this zoonotic disease. The present work aims to describe the tool, its rationale, and the supporting assumptions made to create this accurate and easy-to-use economic estimator.

## 2. Materials and Methods

### 2.1. Overview

The calculator is based on a modified partial budgeting approach applied at the herd level. The first outcome of interest for the farmer is the total yearly impact of Q fever in his or her herd before any intervention for a given prevalence of Q fever, defined as the yearly production losses due to Q fever (*ProdLoss_QfPrev_*). The second outcome of the calculator is the 3-year benefit of Q fever vaccination for the herd (*VaccBenefit_QfPrev_*).

*ProdLoss_QfPrev_* is defined as follows: (i) prevaccination according to the prevalence of Q fever (*QfPrev*) prior to any intervention (*QfPrev_BefVacc_)* and (ii) postvaccination according to the expected prevalence of Q fever postvaccination (*QfPrev_AfterVacc_).* The 3-year *VaccBenefit* is then defined as follows:

*VaccBenefit* = *ProdLoss_QfPrevBefVacc_* − *ProdLoss_QfPrevAfterVacc_* − *CostVaccine*, (1)

where

*CostVaccine* is the total cost of herd vaccination for the 3-year period.

*QfPrev_BefVacc_* is considered constant for the 3-year analysis period, considering that there is no intervention by farmers and that circulation within the herd leads to constant prevalence (infected culled cows replaced by infected heifers). *QfPrev_AfterVacc_* decreases each year due to herd dynamics (infected culled cows replaced by vaccinated heifers).

The vaccination programme was carried out using Coxevac^®^ (Ceva, Libourne, France) with the following protocol: all cattle above 3 months of age were vaccinated every year, i.e., 2 injections for all cattle above 3 months of age in the first year plus 2 injections for 3- to 12-month-old heifers in years 2 and 3 and an annual booster for other cattle from year 2 onwards.

### 2.2. Production Losses for a Herd with Q Fever

The production losses *ProdLoss_QfPrev_* are defined as follows:

*ProdLoss_QfPrev_* = *CostAbort_QfPrev_* + *CostRFM_QfPrev_* + *CostMet_QfPrev_* + *CostAI_QfPrev_* + *CostCCI_QfPrev_*, (2)

where

*CostAbort_QfPrev_* is the cost of extra abortions (*Abort*) due to Q fever for *QfPrev* compared to the situation with no Q fever;

*CostRFM_QfPrev_* is the cost of extra RFMs due to Q fever for *QfPrev* compared to the situation with no Q fever;

*CostMet_QfPrev_* is the cost of extra purulent vaginal discharge and endometritis (metritis-*Met*) due to Q fever for *QfPrev* compared to the situation with no Q fever;

*CostAI_QfPrev_* is the cost of extra artificial insemination (*AI*) due to Q fever for *QfPrev* compared to the situation with no Q fever; 

*CostCCI_QfPrev_* is the cost of extra days of calving-conception interval (*CCI*) due to Q fever for *QfPrev* compared to the situation with no Q fever.

### 2.3. Yearly Prevalence of Q Fever in the Herd

*QfPrev* is considered the main indicator of the herd infection level. The economic impact of prevaccination and the benefits of vaccination are linked to *QfPrev* and the decrease in this value due to vaccination, respectively. Due to herd dynamics and the culling of cows with Q fever that are replaced with vaccinated disease-free heifers, the value of *QfPrev* decreases postvaccination. The model does not consider that cows with Q fever are at greater risk of being culled.

Although the model is static, *QfPrev* is calculated yearly (*QfPrev_Yx_*) based on the mean between *QfPrev* at the beginning of the year (*QfPrev_StartY_*) and that at the end of the year (*QfPrev_EndY_*), as indicated in the following:

*QfPrev_Yx_* = *Avg* (*QfPrev_StartYx_*; *QfPrev_EndYx_*). (3)

*QfPrev_StartY_*_1_ is an input parameter of the final calculator, representing the prevalence of the herd prior to vaccination. The full vaccination of the herd starts at the beginning of year 1, and boosters are administered yearly. The clinical protection brought about by vaccination is modelled in the present work through a change in *QfPrev_Yx_*, even if the prevention of infection through vaccination is not evidenced: this structure of the economic model is in accordance with Equation (2), where the economic consequences of Q fever linked to clinical outcomes are modelled through *QfPrev_Yx_*.

The calculation of *QfPrev_Yx_* (Equation (3)) is based on a simplified compartmental population approach for a fixed-size herd. The first subpopulation is the Q fever-free heifers joining the in-milk cow herd. This subpopulation is considered to remain Q-fever-free due to the vaccination, and the subpopulation size is calculated based on the culling rate of the herd (*CullRate*), assuming equal culling and replacement rates (fixed herd size). The second subpopulation is composed of culled cows that leave the herd based on farmer criteria: these cows can have and not have Q fever, with a share equal to *QfPrev_StartYx_*, assuming that random culling occurs among the cows (no consideration of Q fever status for culling decisions). The third and final subpopulation is composed of cows remaining in the herd for the whole year, with a share between cows with and without Q fever equal to *QfPrev_StartYx_*. Combining the 3 subpopulations leads to the following:

*QfPrev_EndYx_* = *(QfPrev_StartYx_* − *(QfPrev_StartYx_* * *(1 − CullRate)))*. (4)

Equation (4) is applied independently for years 1, 2, and 3 via Equation (5), and the results are combined in Equation (3) to obtain the mean prevalence of Q fever in the herd for each year.

*QfPrev*_*StartYx*+1_ = *QfPrev_EndYx_*. (5)

### 2.4. Disease Impact before Vaccination

As detailed in Equation (6), *CostAbort_QfPrev_* is based on the number of cows with Q fever (HerdSize * QfPrev_StartY__1_) and the number of heifers with Q fever (HerdSize * QfPrev_StartY__1_ * *CullRate* * *Mitigation_Heifers_*), multiplied by the difference in abortion risk in those with Q fever compared to those without Q fever (*AbortRate_NoQf_* * (*RR_AbortIfQf_* − 1)).

*CostAbort_QfPrev_* = (HerdSize * QfPrev_*StartY*1_ + HerdSize * QfPrev_*StartY*1_ * *CullRate* * *Mitigation_Heifers_*) * (*AbortRate_NoQf_* * (*RR_AbortIfQf_* − 1))* *UnitCost_Abort_*, (6)

where

*AbortRate_NoQf_* is the abortion rate of animals with no Q fever;

*RR_AbortIfQf_* is the relative risk (RR) for abortion in animals with Q fever compared to those without Q fever;

*Mitigation_Heifers_* is the mitigation coefficient for heifers to account for the expected lower prevalence of Q fever in heifers than in cows in a given herd; 

*UnitCost_Abort_* is the unit cost of abortion.

Similarly, *CostRFM_QfPrev_* is based on the number of cows with Q fever and the difference in the risk of RFMs in cows with Q fever compared to that in cows without Q fever (Equation (7)):

*CostRFM_QfPrev_* = (*HerdSize* * *QfPrev*_*StartY*1_) * (*RFMRate_NoQf_* * (*RR_RFMIfQf_* − 1)) * *UnitCost_RFM_*, (7)

where

*RFMRate_NoQf_* is the RFM rate for cows with no Q fever;

*RR_RFMIfQf_* is the RR for RFMs in cows with Q fever compared to those without Q fever; 

*UnitCost_RFM_* is the unit cost for RFM treatment.

Next, *CostMet_QfPrev_* is based on the number of cows with Q fever and the difference in the degree of risk for metritis in cows with Q fever compared to cows without Q fever (Equation (8)):

*CostMet_QfPrev_* = (*HerdSize* * *QfPrev*_*StartY*1_) * (*MetRate_NoQf_* * (*RR_MetIfQf_* − 1)) * *UnitCost_Met_*, (8)

where

*MetRate_NoQf_* is the metritis rate in cows with no Q fever;

*RR_MetIfQf_* is the RR for metritis in cows with Q fever compared to those without Q fever; 

*UnitCost_Met_* is the unit cost for metritis treatment.

Finally, *CostAI_QfPrev_* is based on the number of animals with Q fever, including heifers, in terms of abortion as well as the number of extra artificial insemination (AI) procedures in the case of Q fever, as indicated as follows:

*CostAI_QfPrev_* = (*HerdSize* * *QfPrev*_*StartY*1_ + *HerdSize* * *QfPrev*_*StartY*1_ * *CullRate* * *Mitigation_Heifers_*) * Extra*AI_IfQf_* * *UnitCost_AI_*, (9)

where

Extra*AI_IfQf_ i*s the extra service per conception in patients with Q fever compared to patients without Q fever; 

*UnitCost_AI_* is the unit cost for AI.

In addition to this disorder, the literature also shows an association between Q fever and (i) late AI after previous AI [9], (ii) deteriorated first service conception rate (FSCR; [10]), and (iii) calving to conception interval (CCI; [10]). Based on this literature and expert opinion and as detailed in the calibration section, these 3 contributors are included in the component (*CostCCI_QfPrev_*) of Equation (2), as indicated in Equation (10). As a consequence, *CostCCI_QfPrev_* is based on the number of animals (cows and heifers) with Q fever and the additional days for CCI for animals with Q fever, considering extra CCIs based on 3 components. First, extra CCIs associated with late AI after previous AI in cases of Q fever (Extra*CCI_IfQf_LateAI_*) are considered for cows and heifers; second, extra CCIs associated with deteriorated FSCR in cases of Q fever (ExtraCCI*_IfQf_FSCR_*) are applied for cows and heifers; third, extra CCIs directly associated with Q fever (ExtraCCI*_IfQf_DirectQf_*) are included for cows only (Equation (10)).

*CostCCI_QfPrev_* = ((HerdSize * QfPrev_*StartY*1_) * (Extra*CCI_IfQf_LateAI_* + Extra*CCI_IfQf_FSCR_* + Extra*CCI_IfQf_DirectQf_)* + (*HerdSize* * *QfPrev*_*StartY*1_ * *CullRate* * *Mitigation_Heifers_*) * (Extra*CCI_IfQf_LateAI_* + Extra*CCI_IfQf_FSCR_*)) *UnitCost_CCI_, (10)

where

*UnitCost_CCI_* is the unit cost per extra day of CCIs.

### 2.5. Disease Impact of Vaccination and the Cost of Vaccination

The decrease in the value of *QfPrev_Yx_* in the case of vaccination is applied directly to Equations (7)–(10) to assess *CostMet_QfPrev_*, *CostRFM_QfPrev_*, *CostAI_QfPrev_*, and *CostCCI_QfPrev_* for vaccinated herds by replacing *QfPrev_StartY_*_1_ with *QfPrev_Yx_*, respectively. This means that the benefit of vaccination is considered to rely only on the decrease in the prevalence of cows with Q fever as permitted by vaccination.

*CostAbort_QfPrev_* for vaccinated herds also accounts for the decreased risk of abortion for animals with Q fever and who have been vaccinated compared to cows with Q fever and who have not been vaccinated, as detailed in the following:

*CostAbort_QfPrev_* = (*HerdSize* * *QfPrev_Yx_* + *HerdSize* * *QfPrev_Yx_* * *CullRate* * *Mitigation_Heifers_*) * (*AbortRate_NoQf_* * *RR_AbortIfQf_* * (*RR_AbortIfVaccIFQf_* − 1)) * *UnitCost_Abort_*, (11)

where

*RR_AbortIfVaccIfQf_* is the RR for abortion for a cow with Q fever and if vaccinated compared to a non-vaccinated cow.

## 3. Calibration

*HerdSize*, *QfPrev_StartY_*_1_, and *CullRate* are considered fixed in the present work (Table 1) but are adjusted for each farm while using the calculator. To keep the calculator as simple as possible for use in the field, *QfPrev_StartY_*_1_ is used as the main indicator for the Q fever level in the herd, and a half-adjustment for heifers (*Mitigation_Heifers_* = 0.5) is applied to account for the lower-level prevalence of Q fever in heifers than in cows [15,23].

The base prevalence (i.e., not related to Q fever) of abortion, RFMs, and metritis (*AbortRate_NoQf_*, *RFMRate_NoQf_* and *MetRate_NoQf_*) were set at 5%, 4%, and 9%, respectively (Table 1), based on a literature review [24,28]. The values for relative risks or extra service per AI in cases of Q fever (Table 1) are defined based on the literature, as detailed in Appendix A. The values for *ExtraCCI_IfQf___LateAI_* and *ExtraCCI_IfQf___FSCR_* are estimated to be 2 and 3 days, respectively, based on the literature, as detailed in Appendix A.

Unit costs (mean and ranges) are set up by the end users of the calculator. The sample of results reported in the present work is based on expert opinion and grey literature. To avoid any overestimation in the Q fever economic assessment, *UnitCost_Abort_*, *UnitCost_RFM_*, and *UnitCost_Met_* consider only direct costs (production losses, treatment, etc.) and exclude extra labour costs and middle-term consequences for fertility (conception per service and long CCI) that are already considered in other components of the total impact of Q fever (*CostAI_QfPrev_* and *CostCCI_QfPrev_*). This approach is in accordance with epidemiologic studies linking Q fever and service per conception or the CCI, which are not adjusted for the presence of abortion, RFMs, or metritis.

No discount rate is included due to the limited period (3 years) considered for the model. The vaccination cost includes only the vaccine and excludes the labour cost.

## 4. Results and Discussion

### 4.1. Model Highlighting the Cost of Q Fever for the Farmer and the Benefits of Vaccination

The present model was developed to guide farmers’ decisions. This model is not designed to draw general conclusions on Q fever cost components. Through a good understanding of its framework (how it works) and the observation of some results, it remains possible to highlight how the model behaves while avoiding any general extrapolation.

The model shows that abortion and CCI are the two main components of both the economic impact of Q fever and the benefits of the related vaccination (Table 2), regardless of the calibration. Abortion represents 25 to 30% of total costs or vaccination benefits, and changes in the CCI account for 46 to 53%, depending on the model input parameters. Additional AI, RFM, and metritis treatments contributed to 20–25% of the economic impact of Q fever or vaccination benefits (Table 2). This observation (abortion and CCI as the two main components) is in agreement with how the models were built, i.e., with most of the middle-term impact of Q fever on reproduction and with the high-level impact of Q fever on reproduction. These shares of each contributor in the total cost or benefit appear stable even with low or high input parameters.

Table 2 shows that the 3-year total cost of Q fever for 100 cows ranges from EUR 7500 to EUR 23,000 depending on the values used for *QfPrev_StartY_*_1_ and *UnitCost.*

Case 1 of Table 2 represents an average situation of calibration and shows a positive net benefit of vaccination. The breakeven points represent the values of those input parameters for which the vaccination benefit is just above zero (the beginning of positive returns for vaccination). In herds with a moderate or higher prevalence of Q fever, which was simulated here with an initial prevalence (*QfPrev_StartY_*_1_) of 30% or above, a combination of breakeven points for unit costs is EUR 2, EUR 300, EUR 35, EUR 50, and EUR 50 for *UnitCost_CCI_*, *UnitCost_Abort_*, *UnitCost_AI_*, *UnitCost_RFM_*, and *UnitCost_Met_*, respectively (case 2, Table 2). In these situations of a moderate initial prevalence of Q fever, the calculator can be used to better appreciate the benefits of the vaccination, especially depending on the model input parameters. As abortion and CCI are the main contributors and as the results show that the outcomes are highly sensitive to these two components, users must focus on appropriate values for *UnitCost_Abort_* and *UnitCost_CCI_*. *UnitCost_CCI_* has been reported to be non-uniform: its value dramatically changes, especially based on the mean CCI and price of milk [27]. Under the main dairy production system in the EU, the minimum value for *UnitCost_CCI_* should be considered equal to EUR 2, and *UnitCost_CCI_* increases very quickly for herds with a deteriorated CCI or with high costs for milk not produced. Similarly, *UnitCost_Abort_* depends on the genetic level of the herd, the physiologic stage of abortion, the parity of the aborted cow, and the average milk production level of the herd (peak and persistence) [24]. Although its influence on the results is limited, *UnitCost_AI_* also highly varies across countries and production systems, including criteria such as the genetic level, the actors involved (farmer or veterinarian, for instance), and the costs of semen or drugs [26].

The results are also highly sensitive to *QfPrev_StartY_*_1_, with, for instance, high-level benefits in the case of *QfPrev_StartY_*_1_ = 40%, regardless of the value of *UnitCost* (cases 3 and 4, Table 2). In herds with a very limited degree of circulation of Q fever, simulated here with an initial prevalence (*QfPrev_StartY_*_1_) of 20% (case 5), a combination of breakeven points for unit costs is EUR 3.5, EUR 400, EUR 40, EUR 60, and EUR 60 for *UnitCost_CCI_*, *UnitCost_Abort_*, *UnitCost_AI_*, *UnitCost_RFM_*, and *UnitCost_Met_*, respectively. In these herds with low *QfPrev_StartY_*_1_ values, vaccination remains cost-efficient in cases of moderate or high *UnitCost*. This finding shows that the users of the calculator should pay attention to the two key input parameters, *UnitCost_CCI_* and *UnitCost_Abort_*. Moreover, in cases of limited benefits, as suggested by the calculator, vaccination can also be considered a zero-cost or limited-cost insurance calculator to prevent any further deterioration of herd performance.

### 4.2. Partial Budgeting Approach: Adaptations Performed to Scope with Its Limitations

Partial budgeting is no longer considered an appropriate approach for assessing the economic impact of diseases or the benefit of any mitigation measures, especially in dairy production systems. The bias arising from an oversimplified partial budget was discussed in detail previously [25]. This bias arises from (i) the static approach, although dairy production is dynamic and follows a long-term pattern; (ii) the deterministic framework, although both epidemiology and economic stochasticity are observed in the field; (iii) the slicing between the different disorders linked to the studied disease despite multiple interactions between them. Despite these limitations, partial budgeting is preferred to any dynamic stochastic and interactive model here because the first objective is to use a herd-side calculator to support farmer decisions and address the enormous limitations in terms of the epidemiological data available in the studied domain. A cutting-edge methodological model with imprecise calibration is incoherent and can even bias users’ feelings with an overestimation of the robustness of the results they obtain.

One major concern in the economic evaluation of Q fever economic burden or vaccine benefit at the farm level is the precision of epidemiological studies examining the impact of Q fever on animal performance. For instance, in the case of no vaccination, the present model considers the same abortion rate each year after the introduction of Q fever in the herd for the 3-year period of the analysis. The literature shows that after the Q fever-related abortion outbreak in goats, the number of abortions decreased the following year. For cattle, no data are available on the relative epidemiologic pattern of abortion annually. Because Q-fever abortion is more common in goats than in cattle, we assumed that the prevalence of abortion in years 2 and 3 after the outbreak was the same as that in year 1. Similarly, the association between Q fever and endometritis is not consistent [3,6,7,8], and the model considered this association, as indicated in Table 1. The authors believe that this parameter was the most appropriate given the data available in the literature. The results show that endometritis only slightly contributes to the total cost or vaccine benefits, suggesting that this assumption has a very limited impact on the results.

This example also clearly demonstrates that more studies are required to better understand the epidemiology of Q fever in cattle.

The modifications we provide to the partial budget approach allow for a precise simulation of herd dynamics: compartmental modelling year by year for 3 consecutive years succeeds in precisely simulating *QfPrev_Yx_* for the 3 years following vaccination (Table 3). The high-level sensitivity of the results to *QfPrev_StartY_*_1_ (Table 2) and the large change in *QfPrev_Yx_* over time (Table 3) demonstrate the usefulness of compartmental modelling for improving economic model accuracy despite its partial budget basis.

The initial prevalence of Q fever in cows (*QfPrev_StartY_*_1_) is not an indicator that is easy to estimate in the field. The prevalence of Q fever is likely a good proxy for *QfPrev_StartY_*_1_ despite its inability to determine the infectious status of cows. Moreover, the prevalence of this disease is not known among farmers in the field. The prevalence of seropositive cows within milk herds is reported to be 40% under French conditions, ranging between 20 and 60% [29]. The mean prevalence in France was estimated to be 36% in a recent study [5]. For practical use of the calculator in the field, we suggest considering 20% of the minimum value for *QfPrev_StartY_*_1_. Identifying herds whose mean prevalence is less than or greater than 20% is possible because of the use of milk ELISA tests [30]. In the case of a substantial number of abortions in a herd, assuming that the Q fever-related abortion rate is a good proxy of the herd’s Q fever infection status, we suggest using the value of *QfPrev_StartY_*_1_ = 40% (i.e., the approximate mean of French prevalence). Alternatively, the average value for *QfPrev_StartY_*_1_ can reach 30%.

The model framework considers the impact of Q fever on heifers applied to the same percentage (i.e., 20% or 40%) of animals despite evidence of a lower prevalence in heifers than in cows [15,23]. To adjust for this factor, the impact for heifers was considered to be 0.5-fold that of cows (*Mitigation_Heifer_* = 0.5). This assumption enables the use of one value of *QfPrev_StartY_*_1_ for the calibration of the entire model in accordance with the difficulties in assessing *QfPrev_StartY_*_1_ in the field.

### 4.3. Issue of the Reproduction Complex in the Economic Assessment of Dairy Production Outcomes

The economic assessment of Q fever is a typical example of the difficulties that arise when a static model tries to simulate a dynamic and complex system with multiple interactions between the disorders associated with Q fever. First, Q fever simultaneously impacts RFMs, metritis, service performance, and abortion, four items in dynamic interaction, and the quantifications available around Q fever and these four items are very limited.

For *RR_AbortIfQf_*, *RR_RFMIfQf_*, and *RR_MetIfQf_*, the accuracy of the literature seems good (Table 1 and Appendix A). Although only one publication has reported a trend towards decreased degrees of abortion risk in the case of the vaccination of infected cows and heifers, this association is considered in the present economic model [9]. No benefit of the vaccination of infected cows in the reduction of RFMs or metritis is considered in the model due to a lack of evidence in the literature. The risk of overestimation in the Q fever economic assessment is limited in the present work, as only direct costs or treatments for *UnitCost_Abort_, UnitCost_Met_*, *UnitCostRFM*, and *UnitCost_AI_* and middle-term consequences on reproduction performance have been accounted for through the CCI (Appendix A). Similarly, late AI after previous AI [9] and FSCR [10] are considered only through extra CCIs and are associated with two and three CCIs, respectively, as described in Appendix A. Although these calculations are very approximate, the small impact of these two contributors in terms of the CCI when compared to the direct impact of Q fever (+14 days of CCI) shows that the risk of overestimation through these raw calculations is very limited.

As detailed in Appendix A, no data are available on the impact of late AI after previous AI, extra service per conception, or extra CCI when comparing infected herds to noninfected herds, but the opposite association is described when comparing vaccinated populations to nonvaccinated populations [9,10]. Despite this lack of data, the authors consider that any benefit of vaccination can be observed only in the case of the impact of the disease, and the authors extrapolate these three impacts in infected cows and heifers compared to in non-infected cows and heifers. This extrapolation is required to avoid any appropriate estimation of the impact of Q fever when no vaccination is performed, which is an objective of the present work. The lack of consideration of these contributors may lead to severe underestimation.

Building a bioeconomic model requires many trade-offs within its design and calibration. These trade-offs should not be seen as a decrease in the quality of the work or in the precision or robustness of the results; they only represent the best use of the data and knowledge available at this time, considering not only the epidemiological part but also the economic rationale. The concerns previously highlighted about the precision of the model (abortions in case of no vaccination; Q fever and endometritis) or the fact that the slight decrease in milk after vaccination [31] is not considered are in accordance with the assumptions made for the economic part of the model and related to the limitation in the accuracy of the unit costs (Table 1). The vaccination strategy that farmers may develop in the field aims at (i) maximizing the decrease in the bacterial load, (ii) reducing the number of shedders, and (iii) improving reproductive performances. The present model considers that vaccination is occurring at the beginning of the 3-year period and within a herd with Q fever. The available literature does not provide any possibility to consider the bacterial load and number of shedders in the economic assessment in spite of this may influence the economic benefit of vaccination. The present model would benefit from updates as additional information becomes available. 

### 4.4. Focus on Avoiding Overestimation

As detailed above for the population dynamics and for the reproduction complex simulation, the overestimation of the total cost and vaccination benefits is a key driver of the conception and calibration of the estimator. This state of mind also leads to other decisions, such as (i) no cost of labour being included in the model, despite labour costs for the management of the consequences of Q fever possibly being higher than those for vaccination, and (ii) no overculling of cows with Q fever, despite these cows having a higher degree of abortion risk and deteriorated reproductive performance, thus being more likely to be culled. Similarly, the positive effects of vaccination on the public health and health status of neighbouring farms are not accounted for since the model is focused only on the focal farm. Finally, the model considers only 3 years after the start of the vaccination in accordance with the limitations of the model calibration, as previously highlighted, and the difficulties in quantifying the dynamics of Q fever in the herd afterwards. This choice by the authors to ensure the accuracy of the calculator also contributes to vaccination benefit underestimation since we expect greater benefits a few years after the start of the vaccination.

As a tool to be used in the field, the present model is built considering the vaccination of a herd with Q fever already present on the farm, despite vaccination occurring before any infection, as a form of prevention support. This choice to use the vaccine in an infected herd reduces the expected benefits of the vaccine and may underestimate the usefulness of vaccination in Q fever-free herds.

## 5. Conclusions

The economic assessment of Q fever and the potential benefits of vaccination is very challenging due to the scarcity of available data and the dynamic and long-term pattern of dairy production. The improved partial budget model applied to Q fever integrates a simplified yearly compartmental model for a better representation of population dynamism and accounts for the main interactions among the reproduction consequences of Q fever. The model shows a high degree of sensitivity to *QfPrev_StartY_*_1_, *UnitCost_Abort_*, and *UnitCost_CCI_*. The calculator aims to help farmers make decisions at the farm level, but the specific results provided here are not generalizable.

## Figures and Tables

**Table 1:** Model calibration.

	Value (Average and [Range])	Usage in the Model	Reference
*QfPrev_StartY1_* (%)	30 [20–40]	Fixed in the publication. Value to be adjusted to the farm situation by the calculator user	Authors (for the publication)
*HerdSize*	100		
*CullRate* (%)	30		
*UnitCost_Abort_* (EUR)	450 [300–700]	Fixed in the publication. Value to be adjusted to the country or farming system by the calculator users. Possibility of adjusting it to the farm situation	De Vries [24]
*UnitCost_Met_* (EUR)	60 [50–140]		Ferchiou et al. [25]
*UnitCost_RFM_* (EUR)	60 [50–100]		Ferchiou et al. [25]
*UnitCost_AI_* (EUR)	55 [40–65]		Inchaisri et al. [26]
*UnitCost_CCI_* (EUR)	3.5 [2–5]		Meadows et al. [27] and Inchaisri et al. [26]
*Vaccine* (EUR per shot)	8		Expert opinion
*Mitigation_Heifers_* (%)	50	Fixed	Expert opinion
*AbortRate_NoQf_* (%)	5	Fixed	Santos et al. [28] and De Vries [24]
*MetRate_NoQf_* (%)	9	Fixed	
*RFMRate_NoQf_* (%)	4	Fixed	
*RR_RFMIfQf_* (RR)	1.52	Fixed	Ordronneau ^1^ [9]
*RR_MetIfQf_* (RR)	2.5	Fixed	Valla et al. ^1^ [7]
*RR_AbortIfQf_* (RR)	2.25	Fixed	Lopez-Gatius et al. [10] and Ordronneau [9] ^1^
*RR_AbortIfVacIfQf_* (RR)	0.69	Fixed	Ordronneau ^1^ [9]
*ExtraSPC_IfQf_* (number)	0.4	Fixed	Lopez-Gatius et al. ^1^ [10]
*ExtraCCI_IfQf_LateAI_* (number)	2	Fixed	Ordronneau ^1^ [9]
*ExtraCCI_IfQf_FSCR_* (number)	3	Fixed	Lopez-Gatius et al. ^1^ [10]
*ExtraCCI_IfQf_DirectQf_* (number)	14	Fixed	Lopez-Gatius et al. ^1^ [10]

^1^: Details in Appendix A.

**Table 2:** Example of results (total cost of Q fever and vaccination benefits; right) for 5 cases of combinations of input parameters (left). The results are expressed in euros for each year and the 3-year period (bold).

		Value (EUR Per Average Herd of 100 In-Milk Cows)								
		Years	Total	Abortion	CCI	Extra AI	RFMs	Metritis	Vaccination Cost	Benefit Per Year/ Cumulative
**Case 1 (Mean prevalence and mean costs)**		Total cost of Q fever in a herd								
		Y1	3820	970	2018	552	37	243		
Unit cost	Value	Y2	3820	970	2018	552	37	243		
*QfPrev_StartY_*_1_ (%)	30	Y3	3820	970	2018	552	37	243		
*Vaccine* (EUR)	8	**Y1–3**	**11,461**	**2911 (25%)**	**6053 (53%)**	**1656 (14%)**	**112 (1%)**	**729 (6%)**		
*UnitCost_Abort_* (EUR)	450	Benefits of vaccination								
*UnitCost_CCI_* (EUR)	3.5	Y1	1826	692	843	193	13	85	2560	−734
*UnitCost_AI_* (EUR)	40	Y2	3222	887	1665	444	30	196	1520	1702/967
*UnitCost_RFM_* (EUR)	60	Y3	3641	945	1912	520	35	229	1520	2121/3088
*UnitCost_Met_* (EUR)	60	**Y1–3**	**8688**	**2523 (29%)**	**4419 (51%)**	**1157 (13%)**	**78 (1%)**	**509 (6%)**	**5600**	**3088**
**Case 2 (Breakeven costs if mean prevalence)**		Total cost of Q fever in a herd								
		Y1	2526	647	1163	483	31	203		
Unit cost	Value	Y2	2526	647	1163	483	31	203		
*QfPrev_StartY_*_1_ (%)	30	Y3	2526	647	1163	483	31	203		
*Vaccine* (EUR)	8	**Y1–3**	**7578**	**1941 (26%)**	**3488 (46%)**	**1449 (19%)**	**94 (1%)**	**608 (8%)**		
*UnitCost_Abort_* (EUR)	300	Benefits of vaccination								
*UnitCost_CCI_* (EUR)	2	Y1	1197	461	485	169	11	71	2560	−1363
*UnitCost_AI_* (EUR)	35	Y2	2127	591	959	389	25	163	1520	607/−756
*UnitCost_RFM_* (EUR)	50	Y3	2406	630	1102	455	29	191	1520	886/130
*UnitCost_Met_* (EUR)	50	**Y1–3**	**5730**	**1682 (29%)**	**2546 (44%)**	**1013 (18%)**	**65 (1%)**	**425 (7%)**	**5600**	**130**
**Case 3 (High prevalence and mean costs)**		Total cost of Q fever in a herd								
		Y1	5094	1294	2690	736	50	324		
Unit cost	Value	Y2	5094	1294	2690	736	50	324		
*QfPrev_StartY_*_1_ (%)	40	Y3	5094	1294	2690	736	50	324		
*Vaccine* (EUR)	8	**Y1–3**	**15,281**	**3881 (25%)**	**8070 (53%)**	**2208 (14%)**	**149 (1%)**	**972 (6%)**		
*UnitCost_Abort_* (EUR)	450	Benefits of vaccination								
*UnitCost_CCI_* (EUR)	3.5	Y1	2434	922	1124	258	17	113	2560	−126
*UnitCost_AI_* (EUR)	40	Y2	4296	1182	2220	592	40	261	1520	2776/2650
*UnitCost_RFM_* (EUR)	60	Y3	4854	1260	2549	693	47	305	1520	3334/5984
*UnitCost_Met_* (EUR)	60	**Y1–3**	**11,584**	**3364 (29%)**	**5892 (51%)**	**1543 (13%)**	**104 (1%)**	**679 (6%)**	**5600**	**5984**
**Case 4 (High prevalence and high costs)**		Total cost of Q fever in a herd								
		Y1	7694	2013	3830	1012	83	756		
Unit cost	Value	Y2	7694	2013	3830	1012	83	756		
*QfPrev_StartY_*_1_ (%)	40	Y3	7694	2013	3830	1012	83	756		
*Vaccine* (EUR)	8	**Y1–3**	**23,081**	**6038 (26%)**	**11,490 (50%)**	**3036 (13%)**	**250 (1%)**	**2268 (10%)**		
*UnitCost_Abort_* (EUR)	700	Benefits of vaccination								
*UnitCost_CCI_* (EUR)	5	Y1	3683	1434	1601	354	29	265	2560	1123
*UnitCost_AI_* (EUR)	55	Y2	6490	1839	3161	815	67	609	1520	4970/6093
*UnitCost_RFM_* (EUR)	100	Y3	7333	1960	3629	953	78	712	1520	5813/11,906
*UnitCost_Met_* (EUR)	140	**Y1–3**	**17,506**	**5234 (30%)**	**8391 (48%)**	**2122 (12%)**	**174 (1%)**	**1585 (9%)**	**5600**	**11,906**
**Case 5 (Breakeven costs if low prevalence)**		Total cost of Q fever in a herd								
		Y1	2475	647	1345	368	25	162		
Unit cost	Value	Y2	2475	647	1345	368	25	162		
*QfPrev_StartY_*_1_ (%)	20	Y3	2475	647	1345	368	25	162		
*Vaccine* (EUR)	8	**Y1–3**	**7641**	**1941 (25%)**	**4035 (54%)**	**1104 (15%)**	**75 (1%)**	**486 (7%)**		
*UnitCost_Abort_* (EUR)	450	Benefits of vaccination								
*UnitCost_CCI_* (EUR)	3.5	Y1	1166	461	562	129	9	57	2560	−1343
*UnitCost_AI_* (EUR)	40	Y2	2082	591	1110	296	20	130	1520	628/−715
*UnitCost_RFM_* (EUR)	60	Y3	2357	630	1275	346	23	153	1520	907/192
*UnitCost_Met_* (EUR)	60	**Y1–3**	**5792**	**1682 (29%)**	**2946 (51%)**	**772 (14%)**	**52 (1%)**	**340 (6%)**	**5600**	**192**

*QfPrev_Y0_* represents the prevalence of Q fever in the herd before vaccination. Vaccine, *UnitCost_Abort_, UnitCost_RFM_, UnitCost_Met_, UnitCost_AI_* and *UnitCost_CCIet_* are the unit costs for abortion, RFMs, metritis, AI and days of CCI, respectively. % indicates the share of the total costs or benefits.

**Table 3:** Values of *QfPrev_Yx_* obtained with the model depending on *QfPrev_StartY_*_1_.

	Value (%)		
*QfPrev_StartY_*_1_	20	30	40
*QfPrev_Y_*_1_	13.0	19.5	26.0
*QfPrev_Y_*_2_	3.9	5.9	7.8
*QfPrev_Y_*_3_	1.2	1.8	2.3

*QfPrev_Yx_* represents the average prevalence of Q fever in the herd for year x. *QfPrev_StartY_*_1_ represents the prevalence of Q fever in the herd before vaccination.

## Data Availability

The data used in the present work are presented in the tables.

## Appendix A. Epidemiologic Calibration Details

### Appendix A.1. Relative Risk (RR) Associated with Q Fever

The literature on abortion risk in the case of Q fever (Table A1) focuses on cows and heifers with RRs ranging from 2 (abortion rate of 21% vs. 11% [10]) to 2.5 [9,32] and is followed in the present economic assessment, with an average RR of 2.25. To the authors’ knowledge, only one publication [9] has reported a trend (*p* = 0.09) towards decreased abortion risk in the case of the vaccination of infected cows and heifers (RR = 0.69), and this value is retained for calibration (Table 1). The RRs for RFMs (RR = 1.52) and metritis (RR = 2.5) in the case of Q fever are applied for cows only, as described by the unique publication available for each disease [7,9]. No benefit of vaccination in reducing RFMs or metritis in infected cows is considered in the model due to a lack of evidence in the literature.

**Table A1:** Associations between Q fever or vaccination and abortion or reproductive disorders.

		Impact on Cases of Q Fever	Benefits of Q Fever Vaccination
Abortion	Literature ^1^	RR2 = 2 to 2.5 [9,10]	RR ^2^ = 0.69 [0.453–1.06] (*p* = 0.09) [9]
	Model ^1^	RR= 2.25; cows and heifers	RR = 0.69; cows and heifers
RFMs	Literature	RR = 1.52 [95%CI = 1.06–2.19] [9,31]	No [9]
	Model	RR = 1.52; cows only	No ^3^
Metritis		RR = 2.5 [7]	No [9]
		RR = 2.5; cows only	No ^3^
Service per conception	Literature		−0.4 service per conception [10]
	Model	+0.4 service per conception; cows and heifers ^5^	No ^3^
Calving-conception interval (IIC)	Literature		+14 days [10]
	Model	+14 days; cows only ^5^	No ^3^
Late AI after given AI ^4^	Literature	No publication	RR = 0.538 [0.30–0.96] for heifers; *p* > 0.05 for cows [9]
	Model	+2 days extra CCI; cows and heifers ^4^	No ^3^
First Service Conception Rate	Literature	RR ≈ 0.5 [10]	
	Model	+3 days extra CCI; cows and heifer ^5,6^	No ^2^

^1:^ As observed in the literature and as used in the economic model; ^2^: relative risk; ^3^: means that there is no association considered when vaccination occurs in vaccinated animals; the benefits of vaccination remain accounted for through the change in the prevalence of infected cows. ^4^: Second AI occurring between 27 and 90 days after previous AI [9]; ^5^: the association is extrapolated from the benefit observed in animals after vaccination as described in the literature; ^6^: relative risk transformed into extra days of CCI.

### Appendix A.2. Other Reproductive Consequences of Q Fever

The literature reports 0.4 fewer services required per conception in the case of the vaccination of cows and heifers with Q fever [10] and 14 fewer days of the CCI in the case of the vaccination of cows with Q fever [10]. Although no data are available on the impact of Q fever in animals compared to animals without Q fever, the authors believe that any benefit of the vaccination can be observed only in the case of disease impact, and these two associations are applied to animals with Q fever (Table A1).

The last two associations between Q fever and reproductive performance are late AI after previous AI (i.e., AI 27–90 days after previous AI) [9] and changes in the FSCR [10]. To avoid overestimation and to consider the limited quantification of the associations available, these associations are transformed into increases in the CCI. The literature reports late AI after previous AI in vaccinated heifers compared to nonvaccinated heifers with Q fever (RR for AI 27–90 days after previous AI = 0.538 [0.30–0.96]), but no association was observed for cows [9]. The authors also extrapolate this association for infected cows and heifers, even if only demonstrated as a vaccine benefit.

To transform a late AI after previous AI in the case of Q fever into a CCI equivalent, the authors estimate (i) an average conception rate of 0.60, (ii) the minimum difference (to avoid overestimation) in days between the usual reproduction cycle duration (21 days) and the range of delayed AI (27–90 days, as defined by the trial defining the RR), and (iii) the RR to obtain a 2-day extra CCI to late AI after previous AI (0.66 * (27–21 days) * 0.538 = 2 days).

Q fever is also associated with a decrease in the FSCR from 38 to 23% (i.e., an approximately 50% decrease [10]). Considering an average FSCR of 0.70 and RRs of 0.5 and 21 days for the reproduction cycle, the impact of Q fever is transformed into three additional days of CCI ((1 − 0.7) * 0.5 * 21 = 3).
